# Data based model for predicting COVID-19 morbidity and mortality in metropolis

**DOI:** 10.1038/s41598-021-04029-6

**Published:** 2021-12-29

**Authors:** Demian da Silveira Barcellos, Giovane Matheus Kayser Fernandes, Fábio Teodoro de Souza

**Affiliations:** 1 Graduate Program in Urban Management (PPGTU), Pontifical Catholic University of Paraná (PUCPR), Curitiba, Brazil; 2 Department of Computer Science, Pontifical Catholic University of Paraná (PUCPR), Curitiba, Brazil; 3 KU Leuven-Faculty of Economics and Business (FEB), Research Center for Economics and Corporate Sustainability (CEDON), Brussels, Belgium

**Keywords:** Statistics, Atmospheric science, Diseases, Engineering, Mathematics and computing

## Abstract

There is an ongoing need for scientific analysis to help governments and public health authorities make decisions regarding the COVID-19 pandemic. This article presents a methodology based on data mining that can offer support for coping with epidemic diseases. The methodological approach was applied in São Paulo, Rio de Janeiro and Manaus, the cities in Brazil with the most COVID-19 deaths until the first half of 2021. We aimed to predict the evolution of COVID-19 in metropolises and identify air quality and meteorological variables correlated with confirmed cases and deaths. The statistical analyses indicated the most important explanatory environmental variables, while the cluster analyses showed the potential best input variables for the forecasting models. The forecast models were built by two different algorithms and their results have been compared. The relationship between epidemiological and environmental variables was particular to each of the three cities studied. Low solar radiation periods predicted in Manaus can guide managers to likely increase deaths due to COVID-19. In São Paulo, an increase in the mortality rate can be indicated by drought periods. The developed models can predict new cases and deaths by COVID-19 in studied cities. Furthermore, the methodological approach can be applied in other cities and for other epidemic diseases.

## Introduction

The new 2019 coronavirus (COVID-19) is the biggest health challenge that humanity has faced since the Spanish flu outbreak of 1918^1^. Its rapid transmission caused the virus to spread to all continents in a short time. In the absence of drugs or vaccines, non-pharmaceutical interventions were the first strategies governments adopted. Asian countries such as China, Taiwan, Singapore, South Korea and Japan with good experience and training in epidemic management, have employed social isolation and mass screening measures that have succeeded locally in controlling the spread of the virus^2–6^ but with economic and social impacts that cannot yet be fully understood^7^. Even with the worldwide race for developing the vaccine and the search for appropriate treatments for quarantine, lockdown and movement restrictions have been the most adopted strategies by governments. The main reason for these approaches were forecasting models that showed there would be way more deaths without isolation measures^1^. These mathematical models were so important that they changed the direction of the response of some countries. Morbidity and mortality prediction models and a data approach have been essential tools for authorities. For example, in the United Kingdom, Imperial College London modeling made the government change its position, not to adopt any intervention measure, and to enforce quarantine^8^.

Isolation measures seek to delay major outbreaks and level the demand for hospital beds to prevent the collapse of health systems^9^, a tragedy that the world has well observed in the region of Lombardy in Italy^10^. Numerous studies of modeling and data analysis have been carried out to provide support to managers. A first challenge for data scientists has been to predict the evolution of cases and deaths from the disease in different social, environmental and economic contexts^11–21^. A second challenge is to estimate in each region what is the flattening of the contagion curve necessary for the health system not to collapse and what measures are necessary to achieve this hypothetical scenario^1,22^. A third challenge has been understanding which social, economic, and environmental variables correlate with viral dynamics^23–28^. Temperature, for example, was a variable that has been speculated since the virus emerged as important since experts pointed out that low temperatures are more conducive to viral transmission, and subsequently, studies confirmed this hypothesis^23–28^. The rapid response from Asian countries was a global exception in a context where most countries acted too late. This fact demonstrates the lack of global preparation to face diseases where there is an absence of an agile, fast and efficient methodology to provide advice to managers.

This article proposes a methodological approach that has been used to support decision-making in several areas^29–32^, including recently in urban health^33,34^. Although such an approach used infrequently, it is a potential tool in the context of the current health crisis caused by COVID-19 and can contribute to decision-making in facing the virus and other epidemic diseases. This approach makes it possible to identify variables (environmental, climatic, social, etc.) correlated to the disease and allows the prediction of its evolution in a coordinated and agile way with a high degree of accuracy. The scientific community has been working intensively to identify variables related to COVID-19^23–28,35,36^ and to predict the evolution of the disease^11–22^ however, these efforts have taken place separately. This article proposes an approach that integrates the prediction and characterization of explanatory variables quickly and contributes important information to managers. The imminent need to improve preparedness to face outbreaks of epidemic diseases is a legacy that COVID-19 left. The data studied were from the cities of São Paulo (SP), Rio de Janeiro (RJ), and Manaus in Brazil. São Paulo is the most populous in the Southern Hemisphere, and the eighth-largest city globally with more than 12 million inhabitants^37^. Rio de Janeiro is the sixth-largest city in the Americas and the second largest in Brazil, with more than 6 million inhabitants^37^. While Manaus is the seventh-largest city in Brazil with more than 2 million inhabitants and the largest in the country's northern region^37^. In Brazil these three cities had the most deaths from COVID-19 until the first half of 2021. The contributions of this study are:

- Provide a methodology for using data mining as a tool for public health management in metropolises;
- Identify climatic and air quality variables that are correlated with the number of COVID-19 cases and deaths;
- Present and compare the first forecasting models for COVID-19 based on association rules;
- Provide forecasting models of new cases and daily deaths by COVID-19 in SP, RJ and Manaus.

## Study area context

In June of 2021, the USA, India and Brazil, respectively, are the 1st, 2nd and 3rd countries in the world with more cases of COVID-19^38,39^. The spread of the virus occurs in the urban fabric, consequently, large cities are the first focus of outbreaks. Brazil has more than 468 thousand deaths caused by the disease and 16.7 million reported cases ^40^. The city of São Paulo is the epicenter of COVID-19 in Brazil, with more than 828 thousand cases and 32.4 thousand deaths according to official data^39^. In Brazil, the second city with the most deaths from the virus is Rio de Janeiro, with 27.8 thousand deaths, but a much smaller number of confirmed cases, about 354.1 thousand cases^39^. The significant difference in cases is apparently the result of underreporting which is higher in the city of Rio de Janeiro, the lethality rate of the virus (proportion of infected people who died multiplied by 100) in the city is the highest in Brazilian metropolises, about 7.86%^39^, and provides support for this hypothesis. The remaining Brazilian cities have a much lower number of deaths and cases of COVID-19 than São Paulo and Rio de Janeiro. Manaus, for example, the third city in the country with the most deaths, has about 9111 deaths and 183,120 confirmed cases^39^.

In the city of São Paulo, the total number of confirmed cases and deaths of COVID-19 in 2020 surpassed in July all cases and deaths from compulsory notification diseases in 2019. The illness with the highest number of cases and deaths in 2019 was dengue with about 17 thousand cases. These data allow contextualizing the relevance of the new coronavirus in the context of the metropolis of São Paulo. Due to low testing and technical and political problems regarding the counting of cases in Brazil, underreporting is quite high. The number of cases may be up to 12 times greater than that reported as indicated by investigations^41^. The notified cases of SARS in the city of São Paulo at the beginning of July 2020 were already around 22 thousand, about ten times higher than in the whole year of 2019, and the deaths already exceed 7 thousand ^42^, about 30 times more than those killed by the disease in the previous year, which shows that these cases are probably COVID-19.

## Research methodology

### Data acquisition and preparation

The data analyzed are from February 25, 2020 to May 31, 2021. All data used in this research is public. The epidemiological database used is made available in real-time by the state health departments and can be accessed directly through its electronic address (https://brasil.io/covid19/). The isolation index data were obtained only for the city of São Paulo and are made available in real-time by the state government at its website (https://www.saopaulo.sp.gov.br/coronavirus/isolamento/). Climatic data were obtained on the online platform of the Agrometeorological Monitoring System (Agritempo) of the National Institute of Meteorology (INMET) (https://www.agritempo.gov.br/agritempo/produtos.jsp?siglaUF=SP). Air quality data were obtained only for São Paulo from the international platform “Air Quality Historical Data Platform” (https://aqicn.org/data-platform/register/) and the platform is provided by CETESB (State of São Paulo Environmental Company).

The climatic data from three cities are of the automatic Meteorological Stations of INMET (geographic coordinates: SP: 23 K 333498.53 m E, 7405721.27 m S; RJ: 23 K 635041.61 m E, 7482691.62 m S; Manaus: 21 M 172269.15 m E, 9653665.33 m S); these among the stations of Agritempo in these cities have the highest data continuity and number of monitored variables. The São Paulo air quality data are from the CETESB Parque D. Pedro II automatic Air Quality Station (geographic coordinates: 23 K 333573.00 m E; 7394924.00 m S) which among the 18 air quality stations in the city is in a strategic position, which represents the central region of the city is relatively close to the chosen weather station, and with good data continuity.

The variables studied over 462 consecutive days were: new deaths (ND), new confirmed (NC), total confirmed cases (total_conf), total deaths (total_deaths), mortality rate (death_rate—%), isolation index (isol_avg_index—%), minimum (tMin—ºC), average (tAvg—ºC) and maximum (tMax—ºC) temperature, rain (rain_t0—mm), accumulated rain (sum_rain_t1 − today + yesterday; sum_rain_t3 − today + yesterday + the day before yesterday; sum_rain_t5 − today + the previous 4 days; sum_rain_t7 − today + the previous 6 days), drought (drought − days without rain), agricultural drought (dry_drought − days without rain) at 10 mm), wind speed (wind_spe—kmh^−1^), the maximum (dew_pointMax—ºC) and minimum (dew_pointMin—ºC) temperature at which water vapor becomes liquid, minimum atmospheric pressure (atm_pressMin—HpA) and maximum (atm_pressMax—HpA), potential evapotranspiration (pot_evapo—mmd^−1^), real evapotranspiration (real_evapo—mmd^−1^), minimum (urMin—%) and maximum (urMax—%) humidity, soil water availability (soil_wat_avail—%), solar radiation (rad_t0—W/m^2^), solar average radiation (avg_rad_t1 − today average + yesterday; avg_rad_t3 − today average + yesterday + the day before yesterday; avg_rad_t5 − today average + the previous 4 days; avg_rad_t7 − today average + the previous 6 days), month (month), particulate material in the air (pm2.5 and pm10), ozone (o3) and nitrogen dioxide (no2). The air quality variables (pm2.5, pm10, o3 and no2) are normalized in the format of the air quality index (AQI), for each pollutant, using the United States Environmental Protection Agency (USEPA) standard.

In the stage of data preparation, in addition to the unification of the database and standardization, according to the requirements of the statistical and modeling software, the data was discretized, since the modeling software has this requirement. Discretization, which consists of transforming a continuous variable into a categorical one, was done using the statistical tertile (low/0–33%, medium/34–66% and high/67–100%) for each variable.

### Multivariate analyses

Statistical analysis and data grouping have the function of characterizing the database and identifying patterns of association between variables^31^. This step, in addition to guiding the development of the models^30^, can indicate the most important variables from the management point of view. Four analyses were performed (linear correlation, factor analysis, similarity dendrograms and k-means) using the Statistica software (version 7.0, developed by StatSoft). For factor analysis and linear correlation, strong correlations were considered to be positive or negative values greater than or near to 0.6^43^. The similarity dendrograms were constructed from the Euclidean distance. In factor analysis, the principal components were the method used to extract the factors.

### Data modeling

The modeling step consisted of developing predictive models for deaths and new cases on seven consecutive days (t + 1, t + 2, t + 3, t + 4, t + 5, t + 6, t + 7). The two openly available tools used in this stage were CBA (Classification Based on Associations, version 2.0) from the School of Computing, National University of Singapore^44^ and J48, open implementation of the C4.5^45,46^ algorithm in the Weka (Waikato Environment for Knowledge Analysis, version 3.8.5) tool developed by New Zealand University of Waikato^47^. These two modeling tools have similar principles as they use association rules to generate a classifier. However, the essential difference is that J48 is just a classifier expressed as decision trees (set of rules that make the classification of the target variable) ^45^. The algorithm of this tool, C4.5, is one of the most important and widespread in the field of data mining^46^. However, because it is a classifier, a predetermined target is needed to generate the predictive model. While the CBA is a tool that integrates classification and association rules, this allows the analysis of existing standards in the database, the association rules, to also guide the construction of classifiers^44^. The CBA only works with discrete intervals, while the J48 decision tree can work with continuous data, but there is a significant reduction in accuracy, which can reach up to 20%^48^. Therefore, the J48 also opted for the use of the same categorical intervals developed for the CBA.

The classification rules have the format IF (A) / THEN (B) where from an interval (categorical variable) or a set of intervals it is possible to predict (classification rules). Therefore, the rules express an antecedent value (A) and a consequent value (B). So IF “A > 1 THEN → B > 3”. The rules have support (S%) and confidence (C%) that are statistical parameters to filter them. The support (S%) corresponds to the percentage of records, A and B, which were classified correctly, in relation to all records in the database. Accuracy or confidence (C%) indicates the percentage of records that the rule or classifier was correct. The confusion matrix is a table that presents the classification frequencies for each class of the model (true positive, false positive, true negative and false negative). The classifier's accuracy is the sum of the main diagonal (correct classifiers) of the matrix, divided by the total of values and multiplied by 100.

## Results

### Methodological approach

**Figure 1:**
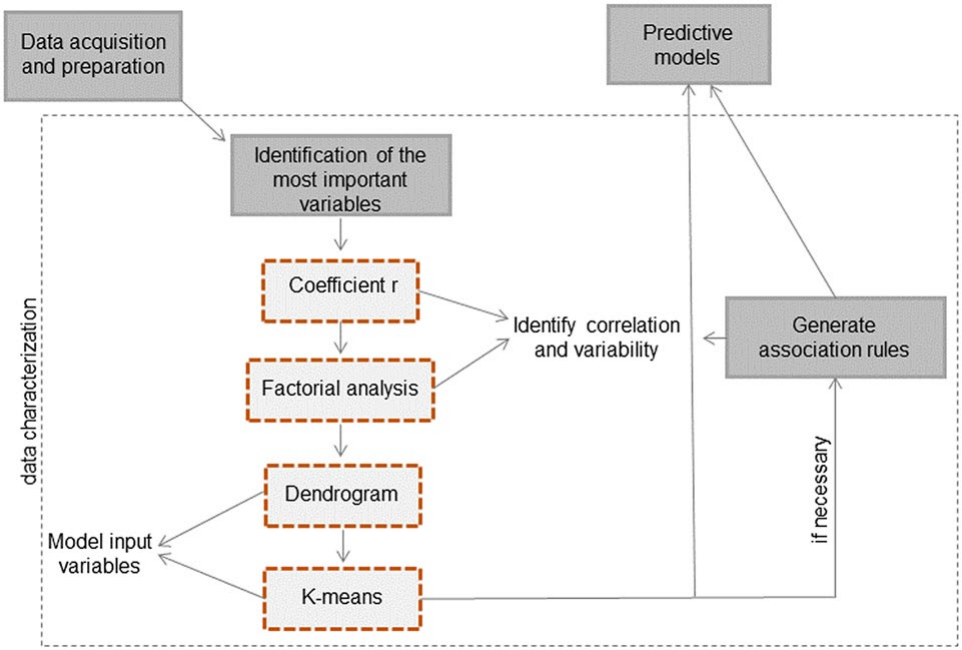
Sequential diagram of the proposed data approach for public health management.

The data approach proposed by this research to support public health in coping with pandemics can be seen in the sequential diagram in Fig. 1. Association rules are also useful tools in discovering patterns between the variables involved, and can be used, if necessary, if those patterns are not yet discovered by statistical analysis and also whether the objective is to explain/estimate the variability of one variable through another. In applying this approach, we do not use association rules because our objective is the prediction and, the statistical analyses were conclusive and revealed the main associations between the variables.

### Relevant variables

The statistical analysis of the linear correlation coefficient showed that for the three cities studied it is challenging to identify a correlation between the epidemiological variables of COVID-19 and environmental variables. In Rio de Janeiro, non-significant linear correlation was identified between environmental and epidemiological variables. In Manaus, the variables, total number of deaths (total_deaths) and confirmed cases (total_conf) presented a significant linear correlation coefficient with the average solar radiation per week (avg_rad_t7) (−0.55 and −0.58 with p < 0.05). In Manaus there was also a significant correlation between new cases (NC) and new deaths (ND) (0.70 with p < 0.05). This correlation also occurred in RJ (0.55 with p < 0.05) and in SP (0.75 with p < 0.05). In SP, the death rate (death_rate) was the epidemiological variable that presented the most significant linear correlation coefficients with the environmental variables. These correlations were with actual evapotranspiration (real_evapo), soil water availability (soil_wat_avail) and agricultural drought (dry_drought)—respectively, −0.57, −0.62 and 0.59 with p < 0.05. In São Paulo, the relationship between mortality rate and average social isolation index was also significant (isol_avg_index), 0.74 with p < 0.05.

**Figure 2:**
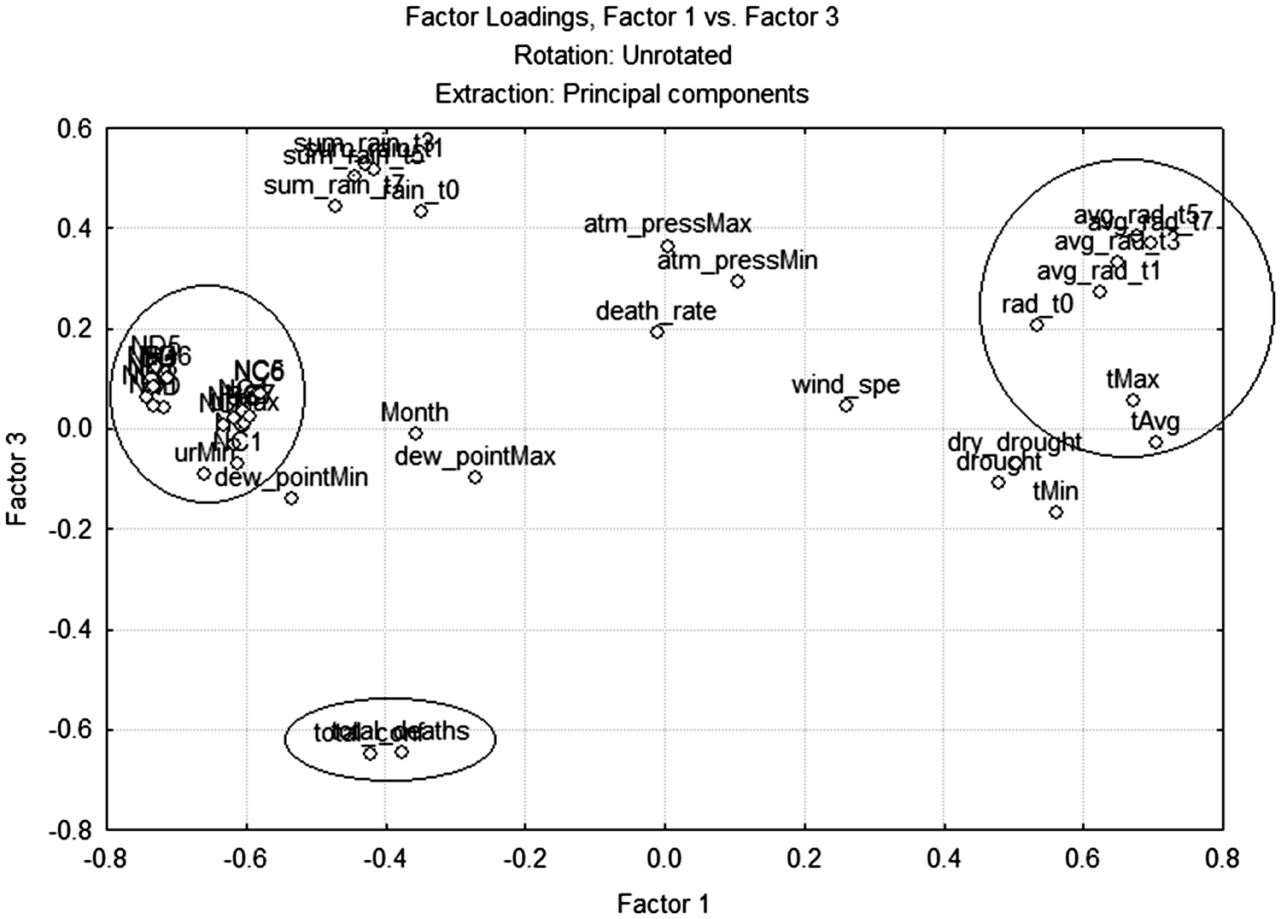
Analysis of the main components in Manaus (generated by the Statistica 7 software—https://statistica.software.informer.com/).

**Figure 3:**
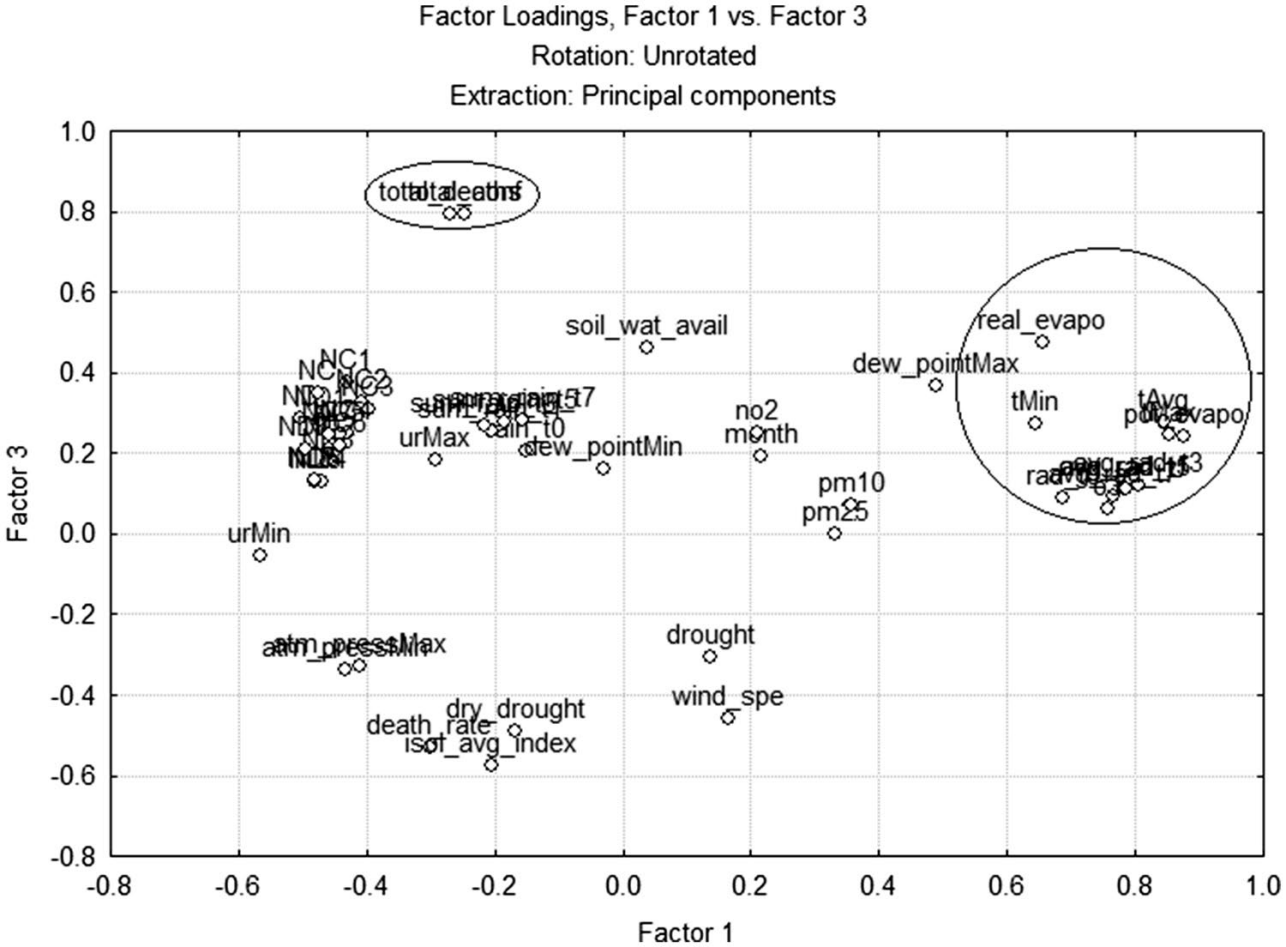
Analysis of the main components in SP(generated by the Statistica 7 software—https://statistica.software.informer.com/).

As in the analysis of linear correlation coefficients, in the factor analysis, it was also difficult to find factor loadings high, greater than 0.60, in the same factor of epidemiological and environmental variables. It was only possible in the city of Manaus, where in factor 1 climatic and epidemiological variables (tAvg, urMin, urMax, avg_rad_t1, t3, t5, t7, NC and ND for the seven consecutive days) had high factor loadings (Fig. 2). In factor 2, none of the variables presented a factor loading greater than 0.6. While in factor 3 the main components are the epidemiological variables (total_deaths and total_conf). In SP, the factor analysis showed some similarities with Manaus, in the sense that there is a large grouping of climatic variables with a high load in factor 1 and that the main components of factor 3 are the same epidemiological variables. However, despite the climatic variables, the main component of factor 1 does not have any epidemiological variable with a high factor loading (Fig. 3). Factor 2 of SP also has climate and air quality variables as main components (soil_wat_avail, urMin, sum_rain_t3, sum_rain_t5, sum_rain_t7 and pm10). While in RJ, factor 1 and 2 have climatic variables as main components and factor 3 does not have relevant factor loading variables.

**Figure 4:**
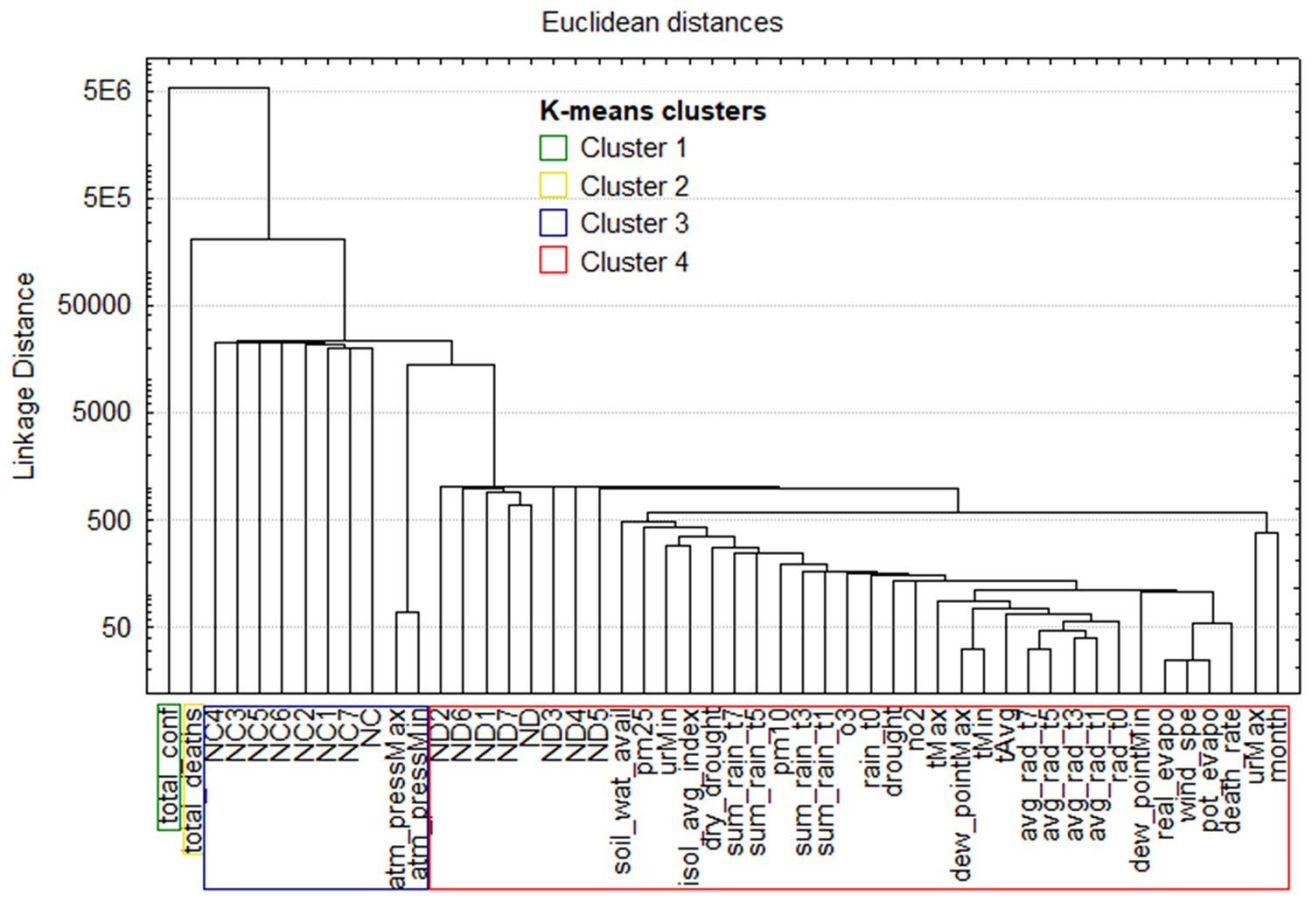
Dendrogram and K-means clusters of SP (generated by the Statistica 7 software—https://statistica.software.informer.com/).

In addition to complementing the characterization of the data initiated with the statistical analyses, the two cluster analyses can indicate which are the best input variables for the generation of models. The similarity dendrogram, constructed with the Euclidean distance between the variables, identifies four same clusters in three cities, is represented in Fig. 4. The first and second clusters are represented by only one variable each, respectively total confirmed (total_conf) and total deaths (total_deaths). In the three cities, the variable new confirmed cases (NC) and its forecast for the seven consecutive days (NC1, NC2, NC3, NC4, NC5, NC6, NC7) were grouped in the third cluster together with atmospheric pressure (atm_pressMax and atm_pressMin), suggesting that the atmospheric pressure can be an input variable in these three cities to predict COVID-19 new cases (NC). All the other variables form the fourth cluster. As one of the goals is to predict the variables ND1, ND2, ND3, ND4, ND5, ND6 and ND7 in the k-means method, we test different numbers of clusters; however, if the option is to group the variables into 4 clusters the three cities present the same clusters visualized by dendrograms (exemplified by Fig. 4).

In k-means, we tested the division into five and six clusters to separate the grouping of target variables from our models, the new deaths (ND). With five clusters, the variables of new deaths (ND, ND1, ND2, ND3, ND4, ND5, ND6 and ND7) remained in the same grouping of variables. What has changed is that the variables of new confirmed cases (NC, NC1, NC2, NC3, NC4, NC5, NC6 and NC7) have separated from atmospheric pressure, forming a single group. It indicates that this grouping is weak and there may be difficulties finding input variables for the new confirmed case models. With six clusters, the variables of new deaths finally formed a smaller grouping together with the maximum relative humidity (urMax), pm2.5 and water availability in the soil (soil_wat_avail), indicating these variables as potential inputs to the models to predict new deaths.

### Predictive models

**Figure 5:**
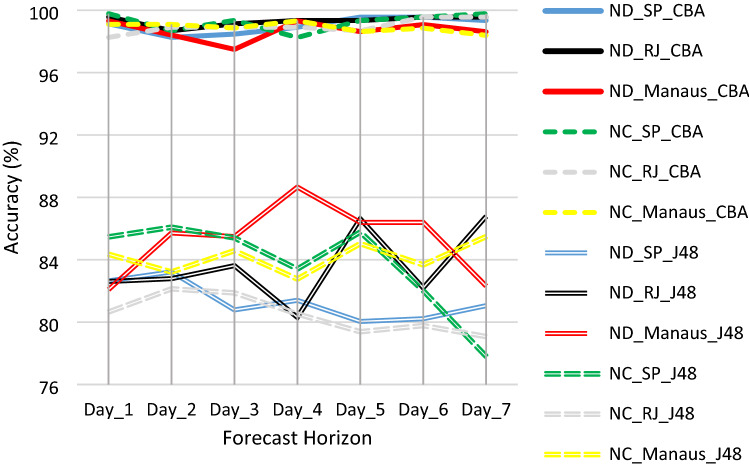
Accuracy comparison of the CBA and J48 prediction models.

Figure 5 shows the accuracy of the two modeling tools used to predict new cases and deaths by COVID-19 in seven consecutive days. The forecasts allow identifying the NC and ND of the next days based on the categorical intervals of the tertiles. Thus it is possible to know for the next few days if the number of new cases (patients) and deaths will correspond to the low (33%), medium (66%) or high (100%) value of the tertiles (NC_SP: 800, 2200 and 8646 patients; NC_RJ: 240, 800 and 7592 patients; NC_Manaus: 200, 400 and 3632 patients; ND_SP: 25, 75 and 378 deaths; ND_RJ: 25, 75 and 307 deaths; ND_Manaus: 5, 15 and 183 deaths). Eighty-four classification models were developed, forty-two by CBA and forty-two by J48—seven predictions for the ND variable and seven for the NC in each city. The results show the best suitability of CBA for the proposed approach. The models developed by J48 showed lower accuracy than CBA’s models (Fig. 5). J48 models' accuracy values range from 77.80% to predict new cases (NC7) in SP to 88.66% to predict new deaths (ND4) in Manaus. In comparison, the accuracy of the CBA models ranged from 97.49% to predict deaths in Manaus (ND3) to 99.78% to predict new cases in SP (NC7).

The CBA generated about a thousand classifiers rules for predicting deaths within a week by city and more about a thousand for predicting new cases of COVID-19. These rules were assessed for their support, accuracy and environmental and epidemiological coherence in their relationship. The choice of classifiers also prioritized those that combined variables related to COVID-19 with environmental variables (climatic and air quality), especially the input variables pointed out by the cluster analysis (atm_pressMax, atm_pressMin, urMax, pm2.5 and soil_wat_avail). A total of twenty-two models were selected to predict new cases and deaths within seven days ahead on three cities studied (Table 1). Managers and authorities in São Paulo, Rio de Janeiro and Manaus can use selected predictive rules as a decision support tool.

**Table 1:** Predictive models generated by the CBA.

New canfirmed (NC)	C%	S%	New deaths (ND)	C%	S%
IF: 800_ < _NC_ < _2200 and 40_ < _isol_avg_index_ < _45 and 15_ < _dew_pointMax_ < _18 THEN→ 800_ < _NC7_SP_ < _2200	81.5	5	IF: soil_wat_avail_ < _55 and ND_ > _75 and tAvg _ < _20 THEN→ ND7_SP_ > _75	100	6.4
IF: 3_<_pot_evapo_<_5 and urMax_>_91 and death_rate_<_9 THEN→ NC2_RJ_<_240 (C%: 81.2, S%: 5.6)	81.2	5.6	IF: soil_wat_avail_ < _55 and ND_ > _75 and May THEN→ ND7_SP_ > _75	100	5.6
IF: NC_<_240 and urMax_>_91 and March THEN→ NC6_RJ_<_240 (C%: 100, S%: 5)	100	5	IF: ND_ > _75 and tMax _ < _25 and 0_ < _sum_rain_t3_ < _5 THEN→ ND7_SP_ > _75	100	6.4
IF: NC_ < _240 and death_rate_ < _9 and March THEN→ NC7_RJ_ < _240	100	6	IF: 36_ < _ urMin _ < _48 and NC_ < _240 and death_rate_ < _9 THEN→ ND4_RJ_ < _25	96.9	6.8
IF: NC_ < _240 and urMax _ > _91 and March THEN→ NC7_RJ_ < _240	100	5.1	IF: NC_ < _240 and death_rate_ < _9 and March THEN→ ND7_RJ_ < _25	100	6
IF: dew_pointMin _>_16 and NC_<_240 and March THEN→ NC7_RJ_<_240	100	5	IF: NC_ < _240 and urMax _ > _91 and March THEN→ ND7_RJ_ < _25	100	5.1
IF: death_rate_ < _5 and total_conf_ > _85000 THEN→ NC2_Manaus_ > _400	100	8	IF: ND_ > _15 and total_deaths_ > _3300 and sum_rain_t5_ > _45 THEN→ ND2_Manaus_ > _15	90.1	9.1
IF: death_rate_ < _5 and total_deaths_ > _3300 and avg_rad_t5_ < _12 THEN→ NC6_Manaus_ > _400	100	6.7	IF: ND_ > _15 and total_deaths_ > _3300 and tMax _ < _31 THEN→ ND1_Manaus_ > _15	93.7	10.2
IF:ND_ > _15 and NC_ > _400 and 5_ < _sum_rain_t3_ < _26 THEN→ NC1_Manaus_ > _400	90.3	6.3	IF: ND_ > _15 and NC_ > _400 and avg_rad_t7_ < _12 THEN→ ND6_Manaus_ > _15	100	10.8
IF:death_rate_ < _5 and total_deaths_ > _3300 and avg_rad_t3_ < _12 THEN→ NC4_Manaus_ > _400	100	6.7	IF: dew_pointMin _ > _21 and ND_ > _15 and NC_ > _400 THEN→ ND7_Manaus_ > _15	93.5	9.9
IF:death_rate_ < _5 and total_deaths_ > _330 and sum_rain_t7_ > _70 THEN→ NC6_Manaus_ > _400	100	6.7	IF: drought_=_0 and ND_ > _15 and NC_ > _400 THEN→ ND6_Manaus_ > _15	94.2	11.2

Forty-two decision trees were generated by J48, seven for new cases and seven for new deaths, within seven days ahead in metropolises studied. Three trees were selected, one for each city, by the accuracy, support, and consistency of the classification rules. Classification trees selected are in Supplementary Material Tables S1, S2 and S3.

## Discussion

The data approach presented and applied in the three Brazilian cities with the most deaths from COVID-19 until the first half of 2021 was first tested only in SP with data from the first wave at the beginning of the pandemic. The modeling of this first application was as successful as that of the second application, presented in this article. However, with the increase in the data series, the variability of epidemiological and environmental variables increased, revealing that the apparent correlation between some variables, in the first hundred days of the pandemic (the first wave), did exist but was not as strong as the trend shown in the first wave. Some of these correlations that drew much attention in the first wave study were the agricultural drought and water availability in the soil, which showed about 0.98 with the total number of deaths and confirmed cases. The increased variability of the data also reduced the support of the CBA classification rules and accuracy of the J48 decision tree compared to the first wave. We noted the need to insert variables derived from other variables into the model that might better explain the complex COVID-19 pandemic phenomenon. These variables reported by other studies as influential in the spread of the virus^24,49–52^ are accumulated precipitation and average solar radiation. In addition to creating new variables, the success of this approach depends on a thorough and skillful data preparation process it is the more important step of data mining^47^. The support of current rules significantly reduced compared to the first wave rules, so we tried to discretize the data using the statistical quartiles and this reduced the rules support in CBA. Therefore, we recommend using statistical tertiles as the best fit for this methodology, as increasing data partitioning will further reduce rule support. Comparative analysis between cities is essential if the objective is to investigate patterns related to the COVID-19 pandemic, which is a complex phenomenon with particularities that reflect the reality of each region. The ambiguity in the relationship between environmental and epidemiological variables in Manaus and SP shows this need, which other studies have highlighted^24,52^.

The proposed methodology (Fig. 1) can be used to face the COVID-19 pandemic and other important diseases such as dengue, malaria and different generations of influenza. This approach is agile, efficient and can quickly provide assistance for coping with these diseases, with an integrated perspective capable of covering the main issues that have been the subject of quantitative research on COVID-19. Therefore, it can be an essential support tool for public health authorities and governments to understand the variables correlated with epidemics and predict new cases and deaths to prepare health systems and control measures. The approach has a reliability of support and forecasting proportional to the reliability of the data. Therefore, comprehensive tracking initiatives and assiduity and authenticity in notifications are essential. This is a challenge for developing countries as Brazil, which have high underreporting documented^41^.

The relationship between epidemiological and environmental variables is quite particular to each of the three cities studied. Correlation coefficients close to or above 0.6 between ND and NC in the three cities demonstrate a directly proportional relationship between these variables. The city of Rio de Janeiro did not show any significant relationship between environmental and epidemiological variables, both in the linear and factorial correlation analysis. A hypothesis that can support this fact is the high rate of underreporting in this city, which, as already mentioned, has higher mortality rates due to COVID-19 among the Brazilian metropolises^39^. As there is a low notification of confirmed cases and many deaths, the mortality rate is falsely presented as high and may have played an important role in masking the natural variability of epidemiological variables related to COVID-19. The lowest correlation between new deaths (ND) and new cases (NC), only 0.55 in RJ, is another indicator that can point to higher values of underreporting in this city compared to Manaus, with 0.70, and SP, 0.75. The linear correlation coefficient between ND and NC is expected to be significant (greater than 0.60 with p < 0.05) due to the likely proportional relationship between morbidity and mortality. The linear correlation coefficient between NC and ND was higher in the city of SP, which had a lower mortality rate (3.91%) than Manaus (4.98%) and Rio de Janeiro (7.86%)^39^, showing the equivalence of the mortality rate and linear correlation coefficient between NC and ND in the cities studied as likely indicate underreporting. Therefore, this linear correlation coefficient and mortality rate can be considered by managers to measure the underreporting rates of different regions comparatively. With low population tracking and monitoring, any data approach to support public health management will encounter great difficulties.

In Manaus, the weekly average of solar radiation (avg_rad_t7) showed an inversely proportional relationship with the total number of confirmed cases and deaths, suggesting, as in other studies^49–51^, that solar radiation can be an important element to reduce the transmissibility of COVID-19 and the deaths caused by the disease. In the factorial analysis, the averages of solar radiation were shown to be a main component together with relative humidity and new deaths (ND, ND1, ND2, ND3, ND4, ND5, ND6 and ND7) to explain the variability of factor 1 (Fig. 2). Furthermore, the factor loadings of new deaths for the seven consecutive days and relative humidity are inversely proportional to solar radiation averages. This indicates that the greater the incidence of solar radiation, the smaller the new daily deaths caused by COVID-19 and relative humidity levels in the air. In Manaus, due to the high incidence of precipitation and solar radiation throughout practically the entire year, relative humidity presented a relationship directly proportional to new deaths. The correlation coefficient was low but directly proportional in the linear correlation, but factor analysis this relationship was very evident due to the high factor loading of these variables. Thus, relative humidity seems to have an ambiguous relationship with the transmissibility and incidence of death from COVID-19^24,52^. In Manaus, with a very rainy climate, the humidity seems to drive an increase in deaths and transmission, as other studies also showed it^24^, and solar radiation a reduction. Therefore, these results suggest that low solar radiation periods predicted in Manaus can guide managers likely to increase deaths due to COVID-19. While in SP, which rains considerably less than in Manaus, the humidity seems to play the opposite role, reducing transmission and deaths from the virus.

In SP agricultural drought (number of days without precipitation greater than 10 mm), the percentage of water available in the soil and real evapotranspiration showed significant linear correlation coefficients (0.59, −0.62, −0.57 with p < 0.05) with a mortality rate of COVID-19. However, the correlation between mortality rate and water availability in the soil and real evapotranspiration was inversely proportional and directly proportional to the agricultural drought. The relationship of these three meteorological variables with the epidemiological variables indicates that the dry climate is a factor that exacerbates the number of COVID-19 infections and deaths. It was observed in other studies, especially in cities with high population density^24,52^. This fact can be explained by the dryness of the airways, which compromises the nasal function of preventing viruses and bacteria into the human body. Therefore, these results suggest that drought periods predicted in SP can guide managers towards an increase in the mortality rate due to COVID-19.

An epidemiological relationship that draws attention to the data from São Paulo is the directly proportional correlation between the mortality rate and the social isolation index. Based on scientific knowledge, it is expected that higher isolation rates will reduce contagion, hospitalizations and deaths from COVID-19^2–6^. So the relationship between death and isolation is expected to be inversely proportional. This directly proportional correlation may be a reflection of the low population tracking and monitoring in Brazil. Since the resulting reason for the mortality rate ((deaths/confirmed cases) × 100) depends on proper screening of confirmed cases. This hypothesis can be strengthened when it is observed that other correlations between isolation index and epidemiological variables (total_deaths and total_conf) are inversely proportional. This suggests that there is a disparity between underreporting of cases and deaths. Another hypothesis that can explain this directly proportional relationship is that in Brazil isolation increases considerably only when health systems are already saturated. So when more deaths occur, social isolation rates increase.

The analysis of the CBA and J48 predictive models showed the greater suitability of the CBA for predicting morbidity and mortality from COVID-19 in cities. CBA is a tool that has already been widely used to predict phenomena of different nature and variability. The findings of this study are in the direction of many other studies that show the potential of CBA to predict complex phenomena^29,32–34^. However, mainly because of its lower accuracy, J48 was less suitable for predicting the complex pandemic phenomenon of COVID-19. Furthermore, the individual support of the J48 rules is quite low, which also makes it inadequate to predict this pandemic phenomenon because there few rule items in the database to characterize the rule as a pattern. This was observed even in the first modeling, with only the data from the first wave (the first hundred days of the pandemic), and it was maintained even with the increase in variability. A third aspect critical in the generated decision trees is the actionability of these models in real life. Due to the great complexity of the trees (Supplementary Material Tables S1, S2 and S3), an automated system would be needed to collect, process data, and automatically provide forecasts to managers. Therefore, because greater accuracy, greater support of rules and greater ease of use we recommend using only the CBA models as a tool for managers and the application in other cities.

The classification rules discovered by data mining techniques revealed other hidden patterns in the data, which were not evident in the statistical analysis because they are not necessarily linear patterns, as of linear correlation and factorial analysis^32^. This is, for example, the case of accumulated rainfall in Manaus or the average radiation for SP (Table 1), patterns that did not show so much strength in the statistical analysis. However, as the factor analysis, the rules showed that Manaus, among the three cities studied, had the greatest environmental influence on the epidemiological aspects of COVID-19. In this city, more environmental patterns could explain the disease. With the greater availability of CBA’s rules with higher support, we have selected a greater number of rules for Manaus, than RJ and SP, which in the statistical analysis did not demonstrate associative patterns between the epidemiological and environmental variables. The overall average accuracy of the J48 models was also the highest in Manaus displaying this pattern too. However, even in RJ and SP cities, the rules identified hidden patterns in the data and generated valuable knowledge for management. Many other possibilities of environmental and social data could be tried by future studies and attempts to apply this approach as a support tool for public health management. These findings show a relevant association among COVID-19 morbimortality and atmospheric conditions based on outdoor meteorological measured on an urban scale. However, a scientific strategy for future study could consider indoor air measures on a smaller scale inside the hospitals and other clinic variables.

Uncertainty about future outbreaks is a problem in recurrent epidemics and pandemics, especially in developing countries with few resources and poor health systems. An accurate predictive tool, capable of anticipating the levels of hospitalizations and deaths, can be useful for health managers.This work elucidates the temporal patterns of morbidity and mortality by COVID-19 in three Brazilian metropolis. The history of confirmed cases and deaths was organized with meteorological and air quality variables. Multivariate analyses were performed to understand the relationships between the variables involved. Predictive models with high and satisfactory precision were built to predict morbidity and mortality. Forecasting public health conditions is useful for preparing health teams in advance for an outbreak and prevents the system from collapsing. In addition, prior information can optimize the resources invested in COVID-19 or other outbreaks of other urban diseases.

## Supplementary Information

Supplementary Tables.
