# Author Correction: Data based model for predicting COVID-19 morbidity and mortality in metropolis

**DOI:** 10.1038/s41598-022-05234-7

**Published:** 2022-01-13

**Authors:** Demian da Silveira Barcellos, Giovane Matheus Kayser Fernandes, Fábio Teodoro de Souza

**Affiliations:** 1 Graduate Program in Urban Management (PPGTU), Pontifical Catholic University of Paraná (PUCPR), Curitiba, Brazil; 2 Department of Computer Science, Pontifical Catholic University of Paraná (PUCPR), Curitiba, Brazil; 3 KU Leuven-Faculty of Economics and Business (FEB), Research Center for Economics and Corporate Sustainability (CEDON), Brussels, Belgium

Correction to: *Scientific Reports* 10.1038/s41598-021-04029-6, published online 29 December 2021

The original version of this Article contained errors.

In the Results section, under the subheading ‘Predictive models,’

“Thus it is possible to know for the next few days if the number of new cases (patients) and deaths will correspond to the low (33%), medium (66%) or high (100%) value of the tertiles (NC_SP: 220, 800 and 8646 patients; NC_RJ: 240, 800 and 7592 patients; NC_Manaus: 200, 400 and 3632 patients; ND_SP: 25, 75 and 378 deaths; ND_RJ: 50, 70 and 100 deaths; ND_Manaus: 5, 15 and 183 deaths).”

now reads:

“Thus it is possible to know for the next few days if the number of new cases (patients) and deaths will correspond to the low (33%), medium (66%) or high (100%) value of the tertiles (NC_SP: 800, 2200 and 8646 patients; NC_RJ: 240, 800 and 7592 patients; NC_Manaus: 200, 400 and 3632 patients; ND_SP: 25, 75 and 378 deaths; ND_RJ: 25, 75 and 307 deaths; ND_Manaus: 5, 15 and 183 deaths).”

Additionally, Table 1 contained errors under the “New confirmed (NC)”, “C%” and “S%” columns. The predictive model rules for NC_RJ were incorrectly swapped with NC_Manaus.

Incorrect:

**Table:** 

New confirmed (NC)	C%	S%	New deaths (ND)	C%	S%
IF: death_rate_ < _5 and total_deaths_ > _3300 and avg_rad_t3_ < _12 THEN → NC4_RJ_ > _400	100	6.2	IF: soil_wat_avail_ < _55 and ND_ > _75 and May THEN → ND7_SP_ > _75	100	5.6
IF: January THEN → NC4_RJ > _400	100	7.1	IF: ND_ > _75 and tMax _ < _25 and 0_ < _sum_rain_t3_ < _5 THEN → ND7_SP_ > _75	100	6.4
IF: death_rate_ < _5 and total_deaths_ > _3300 and avg_rad_t3_ < _12 THEN → NC4_ RJ_ > _400	100	6.6	IF: NC_ < _240 and urMax _ > _91 and March THEN → ND7_RJ_ < _25	100	5.1

Correct:

**Table:** 

New confirmed (NC)	C%	S%	New deaths (ND)	C%	S%
IF: 3_ < _pot_evapo_ < _5 and urMax_ > _91 and death_rate_ < _9 THEN → NC2_RJ_ < _240	81.2	5.6	IF: soil_wat_avail_ < _55 and ND_ > _75 and May THEN → ND7_SP_ > _75	100	5.6
IF: NC_ < _240 and urMax_ > _91 and March THEN → NC6_RJ_ < _240	100	5	IF: ND_ > _75 and tMax _ < _25 and 0_ < _sum_rain_t3_ < _5 THEN → ND7_SP_ > _75	100	6.4
IF: dew_pointMin _ > _16 and NC_ < _240 and March THEN → NC7_RJ_ < _240	100	5	IF: NC_ < _240 and urMax _ > _91 and March THEN → ND7_RJ_ < _25	100	5.1

The original Article has been corrected.

