# Supplementary material for: Data based model for predicting COVID-19 morbidity and mortality in metropolis
Source: Sci Rep. 2021 Dec 29;11:24491. doi: 10.1038/s41598-021-04029-6 (PMC8716530; doi:10.1038/s41598-021-04029-6)
Supplement: Supplementary file 1 — Supplementary Tables. [file 41598_2021_4029_MOESM1_ESM.docx]

**Supplementary Material Table S1: Manaus forecast**

| Predictive model J48 for ND4 in Manaus |
| --- |
| ND = ND_<_5  \| month = Jan: ND4_<_5 (0.0)  \| month = Feb: ND4_<_5 (0.0)  \| month = Mar: ND4_<_5 (19.0)  \| month = Apr  \| \| dew_pointMax = dew_pointMax_<_24: ND4_>_15 (2.0)  \| \| dew_pointMax = 24_<_dew_pointMax_<_25  \| \| \| drought = drought_>_2: 5_<_ND4_<_15 (1.0)  \| \| \| drought = 0_<_drought_<_2: ND4_<_5 (2.0)  \| \| \| drought = drought_eq_0: 5_<_ND4_<_15 (7.0/2.0)  \| \| dew_pointMax = dew_pointMax_>_25: ND4_<_5 (2.0/1.0)  \| month = May  \| \| total_conf = total_conf_<_35000: 5_<_ND4_<_15 (2.0/1.0)  \| \| total_conf = 35000_<_total_conf_<_85000: ND4_<_5 (0.0)  \| \| total_conf = total_conf_>_85000  \| \| \| drought = drought_>_2: ND4_<_5 (8.0)  \| \| \| drought = 0_<_drought_<_2: ND4_<_5 (2.0/1.0)  \| \| \| drought = drought_eq_0: 5_<_ND4_<_15 (3.0)  \| month = Jun  \| \| avg_rad_t5 = avg_rad_t5_>_15: 5_<_ND4_<_15 (2.0)  \| \| avg_rad_t5 = avg_rad_t5_<_12: 5_<_ND4_<_15 (0.0)  \| \| avg_rad_t5 = 12_<_avg_rad_t5_<_15: ND4_>_15 (4.0/1.0)  \| month = Jul  \| \| dew_pointMax = dew_pointMax_<_24  \| \| \| tAvg = tAvg_>_29: 5_<_ND4_<_15 (3.0/1.0)  \| \| \| tAvg = 28_<_tAvg_<_29: ND4_<_5 (3.0)  \| \| \| tAvg = tAvg_<_28: ND4_<_5 (0.0)  \| \| dew_pointMax = 24_<_dew_pointMax_<_25: 5_<_ND4_<_15 (3.0)  \| \| dew_pointMax = dew_pointMax_>_25: ND4_>_15 (1.0)  \| month = Aug  \| \| drought = drought_>_2  \| \| \| tMin = tMin_>_25  \| \| \| \| NC = NC_<_200: ND4_<_5 (3.0/1.0)  \| \| \| \| NC = 200_<_NC_<_400: 5_<_ND4_<_15 (2.0)  \| \| \| \| NC = NC_>_400: 5_<_ND4_<_15 (0.0)  \| \| \| tMin = 24_<_tMin_<_25: ND4_<_5 (2.0)  \| \| \| tMin = tMin_<_24: 5_<_ND4_<_15 (3.0)  \| \| drought = 0_<_drought_<_2  \| \| \| sum_rain_t3 = sum_rain_t3_<_5: ND4_>_15 (2.0)  \| \| \| sum_rain_t3 = 5_<_sum_rain_t3_<_26: ND4_<_5 (2.0)  \| \| \| sum_rain_t3 = sum_rain_t3_>_26: ND4_<_5 (0.0)  \| \| drought = drought_eq_0: ND4_>_15 (1.0)  \| month = Sep: ND4_<_5 (25.0/6.0)  \| month = Oct  \| \| tAvg = tAvg_>_29: 5_<_ND4_<_15 (1.0)  \| \| tAvg = 28_<_tAvg_<_29  \| \| \| NC = NC_<_200: ND4_<_5 (0.0)  \| \| \| NC = 200_<_NC_<_400: ND4_>_15 (2.0)  \| \| \| NC = NC_>_400: ND4_<_5 (2.0)  \| \| tAvg = tAvg_<_28: 5_<_ND4_<_15 (3.0)  \| month = Nov  \| \| atm_pressMin = atm_pressMin_<_1001  \| \| \| tMax = tMax_>_33  \| \| \| \| tMin = tMin_>_25: 5_<_ND4_<_15 (3.0/1.0)  \| \| \| \| tMin = 24_<_tMin_<_25: ND4_<_5 (0.0)  \| \| \| \| tMin = tMin_<_24: ND4_<_5 (2.0)  \| \| \| tMax = 31_<_tMax_<_33  \| \| \| \| atm_pressMax = atm_pressMax_<_1006_5: 5_<_ND4_<_15 (3.0)  \| \| \| \| atm_pressMax = 1006_5_<_atm_pressMax_<_1008: ND4_>_15 (2.0)  \| \| \| \| atm_pressMax = atm_pressMax_>_1008: 5_<_ND4_<_15 (0.0)  \| \| \| tMax = tMax_<_31: 5_<_ND4_<_15 (2.0)  \| \| atm_pressMin = atm_pressMin_>_1002_5: 5_<_ND4_<_15 (0.0)  \| \| atm_pressMin = 1001_<_atm_pressMin_<_1002_5: ND4_<_5 (2.0)  \| month = Dec  \| \| wind_spe = wind_spe_<_0_8  \| \| \| dew_pointMin = dew_pointMin_>_21: 5_<_ND4_<_15 (2.0)  \| \| \| dew_pointMin = 20_<_dew_pointMin_<_21: ND4_<_5 (2.0)  \| \| \| dew_pointMin = dew_pointMin_<_20: ND4_<_5 (0.0)  \| \| wind_spe = 0_8_<_wind_spe_<_1_3: ND4_<_5 (3.0)  \| \| wind_spe = wind_spe_>_1_3: 5_<_ND4_<_15 (3.0)  ND = 5_<_ND_<_15  \| month = Jan: ND4_>_15 (2.0)  \| month = Feb: 5_<_ND4_<_15 (0.0)  \| month = Mar  \| \| avg_rad_t5 = avg_rad_t5_>_15: 5_<_ND4_<_15 (0.0)  \| \| avg_rad_t5 = avg_rad_t5_<_12: ND4_>_15 (4.0/1.0)  \| \| avg_rad_t5 = 12_<_avg_rad_t5_<_15: 5_<_ND4_<_15 (8.0/1.0)  \| month = Apr  \| \| tMin = tMin_>_25: ND4_<_5 (1.0)  \| \| tMin = 24_<_tMin_<_25  \| \| \| sum_rain_t5 = sum_rain_t5_<_15  \| \| \| \| dew_pointMin = dew_pointMin_>_21: ND4_>_15 (2.0)  \| \| \| \| dew_pointMin = 20_<_dew_pointMin_<_21: 5_<_ND4_<_15 (2.0)  \| \| \| \| dew_pointMin = dew_pointMin_<_20: 5_<_ND4_<_15 (1.0)  \| \| \| sum_rain_t5 = 15_<_sum_rain_t5_<_45  \| \| \| \| wind_spe = wind_spe_<_0_8: 5_<_ND4_<_15 (2.0)  \| \| \| \| wind_spe = 0_8_<_wind_spe_<_1_3  \| \| \| \| \| rad_t0 = rad_t0_>_16: ND4_>_15 (2.0)  \| \| \| \| \| rad_t0 = rad_t0_<_12: 5_<_ND4_<_15 (2.0/1.0)  \| \| \| \| \| rad_t0 = 12_<_rad_t0_<_16: ND4_<_5 (2.0)  \| \| \| \| wind_spe = wind_spe_>_1_3: 5_<_ND4_<_15 (0.0)  \| \| \| sum_rain_t5 = sum_rain_t5_>_45: ND4_>_15 (5.0)  \| \| tMin = tMin_<_24  \| \| \| rad_t0 = rad_t0_>_16  \| \| \| \| deaths_rate = death_rate_<_5: 5_<_ND4_<_15 (1.0)  \| \| \| \| deaths_rate = death_rate_>_5_5: ND4_>_15 (3.0)  \| \| \| \| deaths_rate = 5_<_death_rate_<_5_5: 5_<_ND4_<_15 (2.0)  \| \| \| rad_t0 = rad_t0_<_12: 5_<_ND4_<_15 (5.0)  \| \| \| rad_t0 = 12_<_rad_t0_<_16: ND4_>_15 (1.0)  \| month = May  \| \| total_conf = total_conf_<_35000: ND4_>_15 (4.0)  \| \| total_conf = 35000_<_total_conf_<_85000: ND4_>_15 (0.0)  \| \| total_conf = total_conf_>_85000  \| \| \| atm_pressMin = atm_pressMin_<_1001: ND4_<_5 (0.0)  \| \| \| atm_pressMin = atm_pressMin_>_1002_5: ND4_<_5 (9.0/4.0)  \| \| \| atm_pressMin = 1001_<_atm_pressMin_<_1002_5: 5_<_ND4_<_15 (3.0)  \| month = Jun  \| \| avg_rad_t7 = avg_rad_t7_>_15: ND4_<_5 (3.0)  \| \| avg_rad_t7 = avg_rad_t7_<_12: 5_<_ND4_<_15 (1.0)  \| \| avg_rad_t7 = 12_<_avg_rad_t7_<_15  \| \| \| rad_t0 = rad_t0_>_16: 5_<_ND4_<_15 (2.0)  \| \| \| rad_t0 = rad_t0_<_12: ND4_<_5 (2.0/1.0)  \| \| \| rad_t0 = 12_<_rad_t0_<_16: ND4_>_15 (2.0)  \| month = Jul  \| \| drought = drought_>_2  \| \| \| dew_pointMax = dew_pointMax_<_24: ND4_<_5 (6.0/1.0)  \| \| \| dew_pointMax = 24_<_dew_pointMax_<_25: 5_<_ND4_<_15 (3.0/1.0)  \| \| \| dew_pointMax = dew_pointMax_>_25: ND4_<_5 (0.0)  \| \| drought = 0_<_drought_<_2  \| \| \| atm_pressMin = atm_pressMin_<_1001: 5_<_ND4_<_15 (0.0)  \| \| \| atm_pressMin = atm_pressMin_>_1002_5: 5_<_ND4_<_15 (5.0)  \| \| \| atm_pressMin = 1001_<_atm_pressMin_<_1002_5: ND4_<_5 (3.0/1.0)  \| \| drought = drought_eq_0: ND4_<_5 (1.0)  \| month = Aug  \| \| drought = drought_>_2  \| \| \| atm_pressMin = atm_pressMin_<_1001: ND4_<_5 (0.0)  \| \| \| atm_pressMin = atm_pressMin_>_1002_5: 5_<_ND4_<_15 (2.33/0.33)  \| \| \| atm_pressMin = 1001_<_atm_pressMin_<_1002_5: ND4_<_5 (4.67)  \| \| drought = 0_<_drought_<_2: ND4_>_15 (1.0)  \| \| drought = drought_eq_0: ND4_<_5 (2.0/1.0)  \| month = Sep: ND4_<_5 (3.0)  \| month = Oct  \| \| tMax = tMax_>_33: 5_<_ND4_<_15 (10.0/1.0)  \| \| tMax = 31_<_tMax_<_33  \| \| \| urMin = urMin_<_50: ND4_<_5 (0.0)  \| \| \| urMin = 50_<_urMin_<_57  \| \| \| \| avg_rad_t1 = avg_rad_t1_>_15: ND4_<_5 (0.0)  \| \| \| \| avg_rad_t1 = avg_rad_t1_<_12: ND4_>_15 (2.0)  \| \| \| \| avg_rad_t1 = 12_<_avg_rad_t1_<_15: ND4_<_5 (3.0)  \| \| \| urMin = urMin_>_57: 5_<_ND4_<_15 (2.0)  \| \| tMax = tMax_<_31: ND4_<_5 (1.0)  \| month = Nov  \| \| atm_pressMin = atm_pressMin_<_1001: ND4_<_5 (10.0/3.0)  \| \| atm_pressMin = atm_pressMin_>_1002_5: ND4_<_5 (0.0)  \| \| atm_pressMin = 1001_<_atm_pressMin_<_1002_5: 5_<_ND4_<_15 (2.0)  \| month = Dec  \| \| urMin = urMin_<_50: ND4_<_5 (4.0/1.0)  \| \| urMin = 50_<_urMin_<_57  \| \| \| tMin = tMin_>_25: 5_<_ND4_<_15 (2.0)  \| \| \| tMin = 24_<_tMin_<_25: ND4_>_15 (2.0)  \| \| \| tMin = tMin_<_24: 5_<_ND4_<_15 (6.0/1.0)  \| \| urMin = urMin_>_57: 5_<_ND4_<_15 (4.0)  ND = ND_>_15  \| month = Jan: ND4_>_15 (29.0)  \| month = Feb: ND4_>_15 (28.0)  \| month = Mar  \| \| sum_rain_t7 = sum_rain_t7_<_25: ND4_>_15 (0.0)  \| \| sum_rain_t7 = 25_<_sum_rain_t7_<_70: 5_<_ND4_<_15 (5.0/1.0)  \| \| sum_rain_t7 = sum_rain_t7_>_70: ND4_>_15 (14.0/3.0)  \| month = Apr: 5_<_ND4_<_15 (15.0/4.0)  \| month = May  \| \| tMax = tMax_>_33: 5_<_ND4_<_15 (2.0)  \| \| tMax = 31_<_tMax_<_33  \| \| \| dew_pointMin = dew_pointMin_>_21: ND4_>_15 (11.0)  \| \| \| dew_pointMin = 20_<_dew_pointMin_<_21  \| \| \| \| avg_rad_t7 = avg_rad_t7_>_15: ND4_<_5 (2.0)  \| \| \| \| avg_rad_t7 = avg_rad_t7_<_12: ND4_<_5 (1.0)  \| \| \| \| avg_rad_t7 = 12_<_avg_rad_t7_<_15: ND4_>_15 (3.0)  \| \| \| dew_pointMin = dew_pointMin_<_20: ND4_>_15 (0.0)  \| \| tMax = tMax_<_31: ND4_>_15 (8.0/3.0)  \| month = Jun  \| \| wind_spe = wind_spe_<_0_8: ND4_>_15 (6.0/1.0)  \| \| wind_spe = 0_8_<_wind_spe_<_1_3: ND4_>_15 (5.0/2.0)  \| \| wind_spe = wind_spe_>_1_3: 5_<_ND4_<_15 (3.0)  \| month = Jul: 5_<_ND4_<_15 (3.0)  \| month = Aug  \| \| wind_spe = wind_spe_<_0_8: ND4_<_5 (1.0)  \| \| wind_spe = 0_8_<_wind_spe_<_1_3: ND4_<_5 (2.0)  \| \| wind_spe = wind_spe_>_1_3: ND4_>_15 (3.0/1.0)  \| month = Sep: ND4_<_5 (2.0)  \| month = Oct: 5_<_ND4_<_15 (5.0)  \| month = Nov: ND4_<_5 (4.0/1.0)  \| month = Dec: ND4_>_15 (3.0/1.0) |

**Supplementary Material Table S2: Rio de Janeiro forecast**

| Predictive model J48 for ND7 in Rio de Janeiro |
| --- |
| ND = ND_<_25  \| death_rate = death_rate_>_10  \| \| avg_rad_t7 = avg_rad_t7_<_15  \| \| \| drought = drought_<_1: ND7_<_25 (5.0/1.0)  \| \| \| drought = 1_<_drought_<_6  \| \| \| \| wind_spe = wind_spe_>_1_3: 25_<_ND7_<_75 (4.0)  \| \| \| \| wind_spe = 0_8_<_wind_spe_<_1_3: ND7_<_25 (1.0)  \| \| \| \| wind_spe = wind_spe_<_0_8: ND7_<_25 (3.0/1.0)  \| \| \| drought = drought_>_6: ND7_>_75 (2.0)  \| \| avg_rad_t7 = 15_<_avg_rad_t7_<_19: ND7_<_25 (24.0/4.0)  \| \| avg_rad_t7 = avg_rad_t7_>_19: ND7_<_25 (3.0)  \| death_rate = 9_<_death_rate_<_10  \| \| atm_pressMax = atm_pressMax_<_1013  \| \| \| NC = NC_<_240  \| \| \| \| sum_rain_t7 = sum_rain_t7_<_2  \| \| \| \| \| total_conf = total_conf_<_75000: ND7_<_25 (0.0)  \| \| \| \| \| total_conf = 75000_<_total_conf_<_170000: ND7_<_25 (2.0)  \| \| \| \| \| total_conf = total_conf_>_170000: 25_<_ND7_<_75 (4.0/1.0)  \| \| \| \| sum_rain_t7 = 2_<_sum_rain_t7_<_22: 25_<_ND7_<_75 (4.0)  \| \| \| \| sum_rain_t7 = sum_rain_t7_>_22: ND7_<_25 (13.0/2.0)  \| \| \| NC = 240_<_NC_<_800  \| \| \| \| dew_pointMin = dew_pointMin_<_14: ND7_<_25 (2.0)  \| \| \| \| dew_pointMin = 14_<_dew_pointMin_<_16: 25_<_ND7_<_75 (1.0)  \| \| \| \| dew_pointMin = dew_pointMin_>_16  \| \| \| \| \| dry_drought = dry_drought_<_5  \| \| \| \| \| \| dew_pointMax = dew_pointMax_<_19: 25_<_ND7_<_75 (0.0)  \| \| \| \| \| \| dew_pointMax = 19_<_dew_pointMax_<_21: ND7_>_75 (2.0)  \| \| \| \| \| \| dew_pointMax = dew_pointMax_>_21: 25_<_ND7_<_75 (3.0/1.0)  \| \| \| \| \| dry_drought = 5_<_dry_drought_<_12: ND7_>_75 (4.0)  \| \| \| \| \| dry_drought = dry_drought_>_12: ND7_<_25 (1.0)  \| \| \| NC = NC_>_800: ND7_<_25 (0.0)  \| \| atm_pressMax = 1013_<_atm_pressMax_<_1016: ND7_<_25 (5.0/1.0)  \| \| atm_pressMax = atm_pressMax_>_1016: ND7_>_75 (1.0)  \| death_rate = death_rate_<_9  \| \| soil_wat_avail = soil_wat_avail_>_70: ND7_<_25 (19.0/2.0)  \| \| soil_wat_avail = 50_<_soil_wat_avail_<_70: ND7_<_25 (37.0/3.0)  \| \| soil_wat_avail = soil_wat_avail_<_50  \| \| \| avg_rad_t5 = avg_rad_t5_<_15: ND7_<_25 (4.0)  \| \| \| avg_rad_t5 = 15_<_avg_rad_t5_<_19: 25_<_ND7_<_75 (6.0)  \| \| \| avg_rad_t5 = avg_rad_t5_>_19: ND7_<_25 (2.0/1.0)  ND = 25_<_ND_<_75  \| month = Jan: ND7_>_75 (2.0)  \| month = Feb  \| \| NC = NC_<_240: ND7_<_25 (5.0/2.0)  \| \| NC = 240_<_NC_<_800: 25_<_ND7_<_75 (6.0/1.0)  \| \| NC = NC_>_800: ND7_>_75 (1.0)  \| month = Mar  \| \| sum_rain_t7 = sum_rain_t7_<_2: ND7_>_75 (4.0/1.0)  \| \| sum_rain_t7 = 2_<_sum_rain_t7_<_22: 25_<_ND7_<_75 (0.0)  \| \| sum_rain_t7 = sum_rain_t7_>_22  \| \| \| atm_pressMin = atm_pressMin_>_1011: 25_<_ND7_<_75 (0.0)  \| \| \| atm_pressMin = 1008_<_atm_pressMin_<_1011  \| \| \| \| urMax = urMax_>_91: ND7_<_25 (0.0)  \| \| \| \| urMax = 90_<_urMax_<_91: 25_<_ND7_<_75 (2.0)  \| \| \| \| urMax = urMax_<_90: ND7_<_25 (3.0)  \| \| \| atm_pressMin = atm_pressMin_<_1008: 25_<_ND7_<_75 (7.0/1.0)  \| month = Apr  \| \| dew_pointMin = dew_pointMin_<_14: 25_<_ND7_<_75 (4.0/1.0)  \| \| dew_pointMin = 14_<_dew_pointMin_<_16: ND7_>_75 (1.0)  \| \| dew_pointMin = dew_pointMin_>_16: ND7_<_25 (4.0)  \| month = May  \| \| total_conf = total_conf_<_75000  \| \| \| dew_pointMin = dew_pointMin_<_14  \| \| \| \| avg_rad_t7 = avg_rad_t7_<_15: ND7_>_75 (2.0)  \| \| \| \| avg_rad_t7 = 15_<_avg_rad_t7_<_19: 25_<_ND7_<_75 (3.0)  \| \| \| \| avg_rad_t7 = avg_rad_t7_>_19: 25_<_ND7_<_75 (0.0)  \| \| \| dew_pointMin = 14_<_dew_pointMin_<_16: ND7_>_75 (4.0)  \| \| \| dew_pointMin = dew_pointMin_>_16: 25_<_ND7_<_75 (1.0)  \| \| total_conf = 75000_<_total_conf_<_170000: ND7_>_75 (0.0)  \| \| total_conf = total_conf_>_170000  \| \| \| urMin = urMin_>_48: ND7_<_25 (0.0)  \| \| \| urMin = 36_<_urMin_<_48: ND7_<_25 (2.0)  \| \| \| urMin = urMin_<_36: ND7_>_75 (2.0)  \| month = Jun: 25_<_ND7_<_75 (9.0/2.0)  \| month = Jul: 25_<_ND7_<_75 (19.0/5.0)  \| month = Aug  \| \| dew_pointMin = dew_pointMin_<_14  \| \| \| atm_pressMin = atm_pressMin_>_1011: 25_<_ND7_<_75 (6.0/1.0)  \| \| \| atm_pressMin = 1008_<_atm_pressMin_<_1011: ND7_>_75 (3.0)  \| \| \| atm_pressMin = atm_pressMin_<_1008: 25_<_ND7_<_75 (2.0)  \| \| dew_pointMin = 14_<_dew_pointMin_<_16: ND7_<_25 (2.0)  \| \| dew_pointMin = dew_pointMin_>_16: 25_<_ND7_<_75 (0.0)  \| month = Sep  \| \| atm_pressMax = atm_pressMax_<_1013: 25_<_ND7_<_75 (2.0)  \| \| atm_pressMax = 1013_<_atm_pressMax_<_1016  \| \| \| avg_rad_t1 = avg_rad_t1_<_15: ND7_>_75 (1.0)  \| \| \| avg_rad_t1 = 15_<_avg_rad_t1_<_19: 25_<_ND7_<_75 (2.0/1.0)  \| \| \| avg_rad_t1 = avg_rad_t1_>_19: ND7_<_25 (3.0)  \| \| atm_pressMax = atm_pressMax_>_1016: 25_<_ND7_<_75 (9.0/1.0)  \| month = Oct  \| \| sum_rain_t1 = sum_rain_t1_eq_0: 25_<_ND7_<_75 (10.0)  \| \| sum_rain_t1 = 0_<_sum_rain_t1_<_3: ND7_<_25 (3.0/1.0)  \| \| sum_rain_t1 = sum_rain_t1_>_3  \| \| \| avg_rad_t3 = avg_rad_t3_<_15: 25_<_ND7_<_75 (4.0)  \| \| \| avg_rad_t3 = 15_<_avg_rad_t3_<_19: ND7_<_25 (2.0)  \| \| \| avg_rad_t3 = avg_rad_t3_>_19: 25_<_ND7_<_75 (0.0)  \| month = Nov  \| \| avg_rad_t3 = avg_rad_t3_<_15: ND7_>_75 (3.0)  \| \| avg_rad_t3 = 15_<_avg_rad_t3_<_19: 25_<_ND7_<_75 (1.0)  \| \| avg_rad_t3 = avg_rad_t3_>_19: 25_<_ND7_<_75 (5.0/2.0)  \| month = Dec  \| \| tMax = tMax_<_29: 25_<_ND7_<_75 (3.0)  \| \| tMax = 29_<_tMax_<_32: ND7_>_75 (3.0)  \| \| tMax = tMax_>_32: 25_<_ND7_<_75 (6.0/1.0)  ND = ND_>_75  \| month = Jan: ND7_>_75 (17.0/3.0)  \| month = Feb  \| \| avg_rad_t5 = avg_rad_t5_<_15: 25_<_ND7_<_75 (0.0)  \| \| avg_rad_t5 = 15_<_avg_rad_t5_<_19: ND7_>_75 (3.0)  \| \| avg_rad_t5 = avg_rad_t5_>_19: 25_<_ND7_<_75 (7.0/1.0)  \| month = Mar  \| \| NC = NC_<_240: ND7_>_75 (0.0)  \| \| NC = 240_<_NC_<_800: ND7_<_25 (2.0/1.0)  \| \| NC = NC_>_800: ND7_>_75 (4.0)  \| month = Apr: ND7_>_75 (21.0/2.0)  \| month = May: ND7_>_75 (39.0/5.0)  \| month = Jun  \| \| rad_t0 = rad_t0_<_15: ND7_>_75 (19.0/2.0)  \| \| rad_t0 = 15_<_rad_t0_<_19: 25_<_ND7_<_75 (3.0/1.0)  \| \| rad_t0 = rad_t0_>_19: ND7_>_75 (0.0)  \| month = Jul  \| \| rad_t0 = rad_t0_<_15: 25_<_ND7_<_75 (7.0/3.0)  \| \| rad_t0 = 15_<_rad_t0_<_19: ND7_<_25 (2.0/1.0)  \| \| rad_t0 = rad_t0_>_19: 25_<_ND7_<_75 (0.0)  \| month = Aug  \| \| dew_pointMin = dew_pointMin_<_14: 25_<_ND7_<_75 (5.0)  \| \| dew_pointMin = 14_<_dew_pointMin_<_16: ND7_>_75 (2.0)  \| \| dew_pointMin = dew_pointMin_>_16: 25_<_ND7_<_75 (0.0)  \| month = Sep: 25_<_ND7_<_75 (3.0/1.0)  \| month = Oct: ND7_<_25 (1.0)  \| month = Nov: 25_<_ND7_<_75 (7.0/2.0)  \| month = Dec: ND7_>_75 (9.0/1.0) |

**Supplementary Material Table S3: São Paulo forecast**

| Predictive model J48 for ND2 in São Paulo |
| --- |
| total_conf = total_conf_<_180000  \| deaths_rate = death_rate_<_3_8: ND2_<_25 (29.0/1.0)  \| deaths_rate = death_rate_>_4_5  \| \| dew_pointMax = dew_pointMax_>_18: ND2_<_25 (4.0)  \| \| dew_pointMax = 15_<_dew_pointMax_<_18  \| \| \| avg_rad_t7 = avg_rad_t7_>_22  \| \| \| \| soil_wat_avail = soil_wat_avail_>_85: 25_<_ND2_<_75 (0.0)  \| \| \| \| soil_wat_avail = 55_<_soil_wat_avail_<_85: ND2_<_25 (3.0/1.0)  \| \| \| \| soil_wat_avail = soil_wat_avail_<_55: 25_<_ND2_<_75 (3.0)  \| \| \| avg_rad_t7 = avg_rad_t7_<q_18  \| \| \| \| sum_rain_t1 = sum_rain_t1_>_1  \| \| \| \| \| sum_rain_t3 = sum_rain_t3_>_5: ND2_>_75 (3.0)  \| \| \| \| \| sum_rain_t3 = 0_<_sum_rain_t3_<_5: ND2_<_25 (4.0)  \| \| \| \| \| sum_rain_t3 = sum_rain_t3_eq_0: ND2_<_25 (0.0)  \| \| \| \| sum_rain_t1 = 0_<_sum_rain_t1_<_1: ND2_>_75 (6.0)  \| \| \| \| sum_rain_t1 = sum_rain_t1_eq_0: ND2_>_75 (0.0)  \| \| \| avg_rad_t7 = 18_<_avg_rad_t7_<_22  \| \| \| \| no2 = no2_<_10: ND2_<_25 (2.0)  \| \| \| \| no2 = 10_<_no2_<_15: 25_<_ND2_<_75 (5.0/1.0)  \| \| \| \| no2 = no2_>_15: ND2_<_25 (1.0)  \| \| dew_pointMax = dew_pointMax_<_15  \| \| \| rain_t0 = rain_t0_>_1: ND2_<_25 (1.0)  \| \| \| rain_t0 = 0_<_rain_t0_<_1: ND2_>_75 (14.0/3.0)  \| \| \| rain_t0 = rain_t0_eq_0  \| \| \| \| sum_rain_t3 = sum_rain_t3_>_5  \| \| \| \| \| urMax = urMax_>_90: ND2_<_25 (0.0)  \| \| \| \| \| urMax = 85_<_urMax_<_90: 25_<_ND2_<_75 (2.0)  \| \| \| \| \| urMax = urMax_<_85: ND2_<_25 (2.0)  \| \| \| \| sum_rain_t3 = 0_<_sum_rain_t3_<_5  \| \| \| \| \| rad_t0 = rad_t0_>_22: ND2_<_25 (3.0)  \| \| \| \| \| rad_t0 = rad_t0_<q_18  \| \| \| \| \| \| o3 = 20_<_o3_<_30: ND2_<_25 (3.0)  \| \| \| \| \| \| o3 = o3_<_20: ND2_>_75 (4.0/1.0)  \| \| \| \| \| \| o3 = o3_>_30: ND2_<_25 (0.0)  \| \| \| \| \| rad_t0 = 18_<_rad_t0_<_22: ND2_>_75 (10.0/1.0)  \| \| \| \| sum_rain_t3 = sum_rain_t3_eq_0  \| \| \| \| \| ND = ND_<_25  \| \| \| \| \| \| pm25 = pm25_>_60: ND2_>_75 (9.53/1.0)  \| \| \| \| \| \| pm25 = 45_<_pm25_<_60: ND2_>_75 (3.18)  \| \| \| \| \| \| pm25 = pm25_<_45  \| \| \| \| \| \| \| pm10 = pm10_<_15: 25_<_ND2_<_75 (2.12/0.12)  \| \| \| \| \| \| \| pm10 = 15_<_pm10_<_30: ND2_<_25 (3.18/1.18)  \| \| \| \| \| \| \| pm10 = pm10_>_30: 25_<_ND2_<_75 (0.0)  \| \| \| \| \| ND = 25_<_ND_<_75  \| \| \| \| \| \| avg_rad_t1 = avg_rad_t1_>_22: ND2_>_75 (3.0)  \| \| \| \| \| \| avg_rad_t1 = avg_rad_t1_<q_18: ND2_<_25 (3.0)  \| \| \| \| \| \| avg_rad_t1 = 18_<_avg_rad_t1_<_22: ND2_<_25 (3.0/1.0)  \| \| \| \| \| ND = ND_>_75  \| \| \| \| \| \| no2 = no2_<_10: ND2_>_75 (2.0)  \| \| \| \| \| \| no2 = 10_<_no2_<_15: ND2_>_75 (4.0)  \| \| \| \| \| \| no2 = no2_>_15  \| \| \| \| \| \| \| avg_rad_t7 = avg_rad_t7_>_22: ND2_>_75 (1.0)  \| \| \| \| \| \| \| avg_rad_t7 = avg_rad_t7_<q_18  \| \| \| \| \| \| \| \| atm_pressMin = atm_pressMin_<_923: ND2_>_75 (0.0)  \| \| \| \| \| \| \| \| atm_pressMin = atm_pressMin_>_926  \| \| \| \| \| \| \| \| \| real_evapo = real_evapo_>_3: ND2_<_25 (0.0)  \| \| \| \| \| \| \| \| \| real_evapo = 1_5_<_real_evapo_<_3  \| \| \| \| \| \| \| \| \| \| avg_rad_t1 = avg_rad_t1_>_22: ND2_<_25 (0.0)  \| \| \| \| \| \| \| \| \| \| avg_rad_t1 = avg_rad_t1_<q_18: ND2_<_25 (2.0)  \| \| \| \| \| \| \| \| \| \| avg_rad_t1 = 18_<_avg_rad_t1_<_22: 25_<_ND2_<_75 (2.0)  \| \| \| \| \| \| \| \| \| real_evapo = real_evapo_<_1_5  \| \| \| \| \| \| \| \| \| \| tMin = tMin_>_18: ND2_>_75 (0.0)  \| \| \| \| \| \| \| \| \| \| tMin = 15_<_tMin_<_18: ND2_<_25 (3.0/1.0)  \| \| \| \| \| \| \| \| \| \| tMin = tMin_<_15: ND2_>_75 (5.0/1.0)  \| \| \| \| \| \| \| \| atm_pressMin = 923_<_atm_pressMin_<_926: ND2_>_75 (6.0)  \| \| \| \| \| \| \| avg_rad_t7 = 18_<_avg_rad_t7_<_22: 25_<_ND2_<_75 (2.0)  \| deaths_rate = 3_8_<_death_rate_<_4_5: ND2_<_25 (0.0)  total_conf = 180000_<_total_conf_<_400000  \| deaths_rate = death_rate_<_3_8: 25_<_ND2_<_75 (0.0)  \| deaths_rate = death_rate_>_4_5  \| \| NC = NC_<_800: ND2_>_75 (4.0/1.0)  \| \| NC = 800_<_NC_<_2200: ND2_<_25 (4.0)  \| \| NC = NC_>_2200  \| \| \| dew_pointMin = dew_pointMin_>_13: ND2_>_75 (0.0)  \| \| \| dew_pointMin = 9_<_dew_pointMin_<_13: ND2_<_25 (3.0/1.0)  \| \| \| dew_pointMin = dew_pointMin_<_9  \| \| \| \| tMin = tMin_>_18: ND2_>_75 (0.0)  \| \| \| \| tMin = 15_<_tMin_<_18: 25_<_ND2_<_75 (2.0)  \| \| \| \| tMin = tMin_<_15: ND2_>_75 (7.0/1.0)  \| deaths_rate = 3_8_<_death_rate_<_4_5  \| \| ND = ND_<_25  \| \| \| month = Jan: 25_<_ND2_<_75 (0.0)  \| \| \| month = Feb: 25_<_ND2_<_75 (0.0)  \| \| \| month = Mar: 25_<_ND2_<_75 (0.0)  \| \| \| month = Apr: 25_<_ND2_<_75 (0.0)  \| \| \| month = May: 25_<_ND2_<_75 (0.0)  \| \| \| month = Jun: 25_<_ND2_<_75 (0.0)  \| \| \| month = Jul: 25_<_ND2_<_75 (0.0)  \| \| \| month = Aug  \| \| \| \| o3 = 20_<_o3_<_30: ND2_>_75 (1.0)  \| \| \| \| o3 = o3_<_20: 25_<_ND2_<_75 (3.0)  \| \| \| \| o3 = o3_>_30: ND2_<_25 (2.0/1.0)  \| \| \| month = Sep  \| \| \| \| sum_rain_t7 = sum_rain_t7_>_15: 25_<_ND2_<_75 (0.0)  \| \| \| \| sum_rain_t7 = 0_<_sum_rain_t7_<_15: 25_<_ND2_<_75 (4.0)  \| \| \| \| sum_rain_t7 = sum_rain_t7_eq_0: ND2_>_75 (5.0/2.0)  \| \| \| month = Oct  \| \| \| \| sum_rain_t1 = sum_rain_t1_>_1  \| \| \| \| \| avg_rad_t5 = avg_rad_t5_>_22: 25_<_ND2_<_75 (2.0)  \| \| \| \| \| avg_rad_t5 = avg_rad_t5_<q_18: ND2_<_25 (1.0)  \| \| \| \| \| avg_rad_t5 = 18_<_avg_rad_t5_<_22: ND2_<_25 (5.0)  \| \| \| \| sum_rain_t1 = 0_<_sum_rain_t1_<_1: ND2_<_25 (1.0)  \| \| \| \| sum_rain_t1 = sum_rain_t1_eq_0: 25_<_ND2_<_75 (8.0/2.0)  \| \| \| month = Nov  \| \| \| \| tMin = tMin_>_18: 25_<_ND2_<_75 (6.0/1.0)  \| \| \| \| tMin = 15_<_tMin_<_18: 25_<_ND2_<_75 (3.0)  \| \| \| \| tMin = tMin_<_15  \| \| \| \| \| sum_rain_t3 = sum_rain_t3_>_5: ND2_<_25 (4.0)  \| \| \| \| \| sum_rain_t3 = 0_<_sum_rain_t3_<_5: 25_<_ND2_<_75 (2.0)  \| \| \| \| \| sum_rain_t3 = sum_rain_t3_eq_0: ND2_<_25 (1.0)  \| \| \| month = Dec  \| \| \| \| rain_t0 = rain_t0_>_1: 25_<_ND2_<_75 (9.0/2.0)  \| \| \| \| rain_t0 = 0_<_rain_t0_<_1: 25_<_ND2_<_75 (0.0)  \| \| \| \| rain_t0 = rain_t0_eq_0: ND2_<_25 (3.0)  \| \| ND = 25_<_ND_<_75  \| \| \| sum_rain_t7 = sum_rain_t7_>_15: ND2_<_25 (36.0/16.0)  \| \| \| sum_rain_t7 = 0_<_sum_rain_t7_<_15  \| \| \| \| NC = NC_<_800: 25_<_ND2_<_75 (0.0)  \| \| \| \| NC = 800_<_NC_<_2200  \| \| \| \| \| isol_avg_index = isol_avg_index_<_40: ND2_<_25 (4.0/1.0)  \| \| \| \| \| isol_avg_index = 40_<_isol_avg_index_<_45  \| \| \| \| \| \| sum_rain_t3 = sum_rain_t3_>_5: 25_<_ND2_<_75 (3.0)  \| \| \| \| \| \| sum_rain_t3 = 0_<_sum_rain_t3_<_5  \| \| \| \| \| \| \| drought = drought_<_1: 25_<_ND2_<_75 (0.0)  \| \| \| \| \| \| \| drought = 1_<_drought_<_5: ND2_<_25 (2.0)  \| \| \| \| \| \| \| drought = drought_>_5: 25_<_ND2_<_75 (4.0)  \| \| \| \| \| \| sum_rain_t3 = sum_rain_t3_eq_0: ND2_<_25 (7.0/1.0)  \| \| \| \| \| isol_avg_index = isol_avg_index_>_45: 25_<_ND2_<_75 (1.0)  \| \| \| \| NC = NC_>_2200: 25_<_ND2_<_75 (3.0)  \| \| \| sum_rain_t7 = sum_rain_t7_eq_0: ND2_<_25 (7.0/2.0)  \| \| ND = ND_>_75: 25_<_ND2_<_75 (10.0/1.0)  total_conf = total_conf_>_400000  \| soil_wat_avail = soil_wat_avail_>_85  \| \| rad_t0 = rad_t0_>_22  \| \| \| atm_pressMax = atm_pressMax_<_928  \| \| \| \| no2 = no2_<_10: 25_<_ND2_<_75 (1.14/0.04)  \| \| \| \| no2 = 10_<_no2_<_15: ND2_>_75 (3.43/0.43)  \| \| \| \| no2 = no2_>_15  \| \| \| \| \| isol_avg_index = isol_avg_index_<_40: 25_<_ND2_<_75 (19.57/4.0)  \| \| \| \| \| isol_avg_index = 40_<_isol_avg_index_<_45: ND2_<_25 (2.86)  \| \| \| \| \| isol_avg_index = isol_avg_index_>_45: 25_<_ND2_<_75 (5.0/1.0)  \| \| \| atm_pressMax = 928_<_atm_pressMax_<_930: 25_<_ND2_<_75 (3.0/1.0)  \| \| \| atm_pressMax = atm_pressMax_>_930: ND2_<_25 (2.0/1.0)  \| \| rad_t0 = rad_t0_<q_18  \| \| \| urMin = urMin_>_45: ND2_<_25 (17.0/7.0)  \| \| \| urMin = 35_<_urMin_<_45: 25_<_ND2_<_75 (2.0/1.0)  \| \| \| urMin = urMin_<_35: ND2_<_25 (0.0)  \| \| rad_t0 = 18_<_rad_t0_<_22  \| \| \| rain_t0 = rain_t0_>_1: 25_<_ND2_<_75 (7.0/2.0)  \| \| \| rain_t0 = 0_<_rain_t0_<_1: ND2_>_75 (3.0)  \| \| \| rain_t0 = rain_t0_eq_0: ND2_>_75 (1.0)  \| soil_wat_avail = 55_<_soil_wat_avail_<_85  \| \| month = Jan  \| \| \| pm25 = pm25_>_60  \| \| \| \| sum_rain_t3 = sum_rain_t3_>_5: ND2_>_75 (2.0)  \| \| \| \| sum_rain_t3 = 0_<_sum_rain_t3_<_5: ND2_<_25 (2.0)  \| \| \| \| sum_rain_t3 = sum_rain_t3_eq_0: ND2_<_25 (0.0)  \| \| \| pm25 = 45_<_pm25_<_60: 25_<_ND2_<_75 (4.0)  \| \| \| pm25 = pm25_<_45: ND2_<_25 (2.0)  \| \| month = Feb: 25_<_ND2_<_75 (3.0/1.0)  \| \| month = Mar: ND2_>_75 (11.0/1.0)  \| \| month = Apr: ND2_>_75 (25.0/8.0)  \| \| month = May  \| \| \| isol_avg_index = isol_avg_index_<_40: ND2_>_75 (16.0/3.0)  \| \| \| isol_avg_index = 40_<_isol_avg_index_<_45: 25_<_ND2_<_75 (3.0/1.0)  \| \| \| isol_avg_index = isol_avg_index_>_45: ND2_>_75 (3.0)  \| \| month = Jun: ND2_>_75 (0.0)  \| \| month = Jul: ND2_>_75 (0.0)  \| \| month = Aug: ND2_>_75 (0.0)  \| \| month = Sep: ND2_>_75 (0.0)  \| \| month = Oct: ND2_>_75 (0.0)  \| \| month = Nov: ND2_>_75 (0.0)  \| \| month = Dec: ND2_>_75 (0.0)  \| soil_wat_avail = soil_wat_avail_<_55  \| \| rad_t0 = rad_t0_>_22: ND2_>_75 (1.0)  \| \| rad_t0 = rad_t0_<q_18  \| \| \| urMin = urMin_>_45: ND2_<_25 (2.0)  \| \| \| urMin = 35_<_urMin_<_45: 25_<_ND2_<_75 (3.0/1.0)  \| \| \| urMin = urMin_<_35: ND2_<_25 (0.0)  \| \| rad_t0 = 18_<_rad_t0_<_22: ND2_>_75 (5.0) |
